# Clinicopathological analysis of histological variants of ameloblastoma in a suburban Nigerian population

**DOI:** 10.1186/1746-160X-2-42

**Published:** 2006-11-24

**Authors:** Kehinde E Adebiyi, Vincent I Ugboko, Ganiat O Omoniyi-Esan, Kizito C Ndukwe, Fadekemi O Oginni

**Affiliations:** 1 Department of Oral/Maxillofacial Surgery and Oral Pathology, Obafemi Awolowo University, Ile Ife, Nigeria; 2 Department of Morbid Anatomy and Forensic Medicine, Obafemi Awolowo University, Ile Ife, Nigeria

## Abstract

**Background:**

This study was carried out to establish the relative incidence and provide clinico-pathologic information on the various histological types of ameloblastoma seen at the Obafemi Awolowo University Teaching Hospital complex, Ile-Ife in order to provide a baseline data which will be of significance to the pathologist and clinician.

**Methods:**

Clinico-pathologic data on a total of 77 histologically diagnosed cases of ameloblastoma archieved at the Obafemi Awolowo University Teaching Hospital Complex, Ile-Ife over a 15 year period were obtained and analysed descriptively.

**Results:**

Follicular ameloblastoma was the most common histological type (50 cases, 64.9%), followed by plexiform ameloblastoma (10 cases, 13.0%). 4 (5.2%) cases of desmoplastic and 3 (3.9%) cases of acanthomatous ameloblastoma were seen while the basal cell variant accounted for 2 (2.6%) cases. Only 1 case of the unicystic type was seen. Some of the 77 cases presented as a mixture of two or more histological types. Ameloblastoma occurred over an age range of 11 to 70 years with a peak age incidence in the 3^rd ^decade.

**Conclusion:**

This study provides a baseline data on variants of ameloblastoma as obtained in a suburban Nigerian population. Since variants of ameloblastoma differ in biologic behaviour, the data collected in this study provides clinicopathologic information which is of significance to the pathologist and clinician.

## Background

Ameloblastoma is a neoplasm of odontogenic epithelium, especially of enamel organ-type tissue that has not undergone differentiation to the point of hard tissue formation [1]. It generally occurs in bone, and it has been postulated that the epithelium of origin is derived from one of the following sources: (1) cell rests of the enamel organ, (2) epithelium of odontogenic cysts, (3) disturbances of the developing enamel organ, (4) basal cells of the surface epithelium or (5) heterotropic epithelium in other parts of the body [2]. The theory of an odontogenic origin for the ameloblastoma is supported clinically by the tumour's common occurrence in the tooth bearing area and is further reinforced by the finding of Spouge that one in every three such tumours are mural proliferations in intimate association with the reduced anamel-forming epithelium of dentigerous cysts [3].

In the World Health Organisation (WHO) histological typing of odontogenic tumours [4], ameloblastoma was classified as belonging to the group of lesions in which there is odontogenic epithelium without morphorlically identifiable odontogenic ectomesenchyme. Recently there has been substantial changes in the section on ameloblastoma, some newly recorgnised odontogenic tumours have been added and some lesions previously designated have been moved to another part of the classification or merged into different subgroups.

Amongst the ameloblastomas, there is now more detailed reference to the unicystic variety because both the surgical management and prognosis of these lesions are significantly different from that of other ameloblastomas. Also of note are the desmoplastic ameloblastoma and the keratoameloblastoma. The squamous odontogenic tumour has become accepted as a distinctive lesion rather than a variant of ameloblastoma. Although it has an infiltrative pattern of growth, most cases respond to curettage, and recurrence is rare.

This study was carried out to establish the relative incidence and provide clinico-pathologic information on the various histological types of ameloblastoma seen at the Obafemi Awolowo University Teaching Hospital complex, Ile-Ife over a 15 year period in order to provide a baseline data which will be of significance to the pathologist and clinician.

## Materials and methods

Biopsy records of all histologically diagnosed cases of ameloblastoma during the period from 1990 to 2004 inclusive were retrieved from the files of the biopsy service of the Department of Morbid Anatomy and Forensic Medicine and that of Oral Pathology of the Obafemi Awolowo University Teaching Hospital complex, Ile-Ife. 79 cases of ameloblastoma were extracted for detailed analysis. Haematoxylin and eosin stained sections of the ameloblastomas were retrieved and reviewed in order to reconfirm the diagnosis and where necessary, revise the diagnosis in light of available clinical and histological details and the WHO histological typing of odontogenic tumours[4]. After review, 77 of the 79 cases were confirmed as ameloblastomas and were categorised into different histological types based on the presenting histological features. Data on incidence, age, sex and site of lesions were analysed descriptively for the various variants of ameloblastoma.

## Results

A total of 79 lesions of the oral cavity and jaws were diagnosed as ameloblastoma between January 1990 and December 2004. Of these, 77 cases satisfied the histological criteria for ameloblastoma, some of them being a mixture of two or more histological types. They were categorized into ten histological subtypes (Table 1). Follicular ameloblastoma was the most common histological type (50 cases, 64.9%), followed by plexiform ameloblastoma (10 cases, 13.0%) and desmoplastic ameloblastoma (4 cases, 5.2%). 3 (3.9%) cases of acanthomatous ameloblastoma were seen while the basal cell variant accounted for only 2 (2.6%) cases. Only 1 case (1.3%) of unicystic ameloblastoma was seen. Ameloblastoma occurred over an age range of 11 to 70 years (Table 1) with a peak age incidence in the 3^rd ^decade.

**Table 1:** Histological type/Age group of patient cross-tabulation

	**Age group of patients (years)**						
**Histological type**	**11–20**	**21–30**	**31–40**	**41–50**	**51–60**	**61–70**	**Total (%)**
Follicular	11	22	11	4	1	1	50 (64.9)
Plexiform	2	3	1	0	2	2	10 (13.0)
Acanthomatous	0	0	0	0	1	2	3 (3.9)
Basal cell	0	0	2	0	0	0	2 (2.6)
Desmoplastic	0	1	3	0	0	0	4 (5.2)
Unicystic	0	0	1	0	0	0	1 (1.3)
Cystic/follicular	1	2	0	0	0	0	3 (3.9)
Follicular/desmoplastic	0	2	0	0	0	0	2 (2.6)
Follicular/acanthomatous	0	0	0	1	0	0	1 (1.3)
Follicular/acanthomatous/cystic	0	1	0	0	0	0	1 (1.3)
**Total**	14	31	18	5	4	5	77 (100)

**Figure 1:**
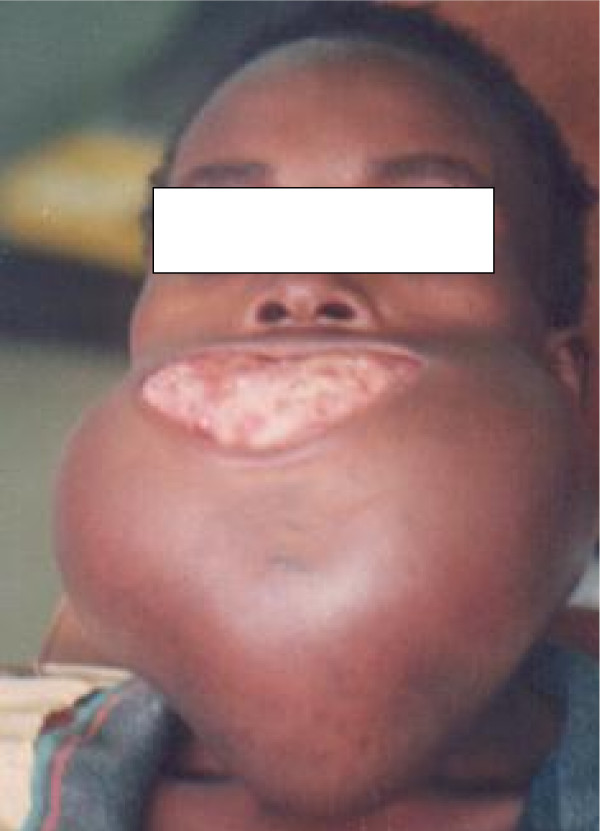
Clinical appearance of a case of ameloblastoma of the mandible.

Follicular ameloblastoma showed equal gender distribution (Table 2). However all the other histological subtypes with the exception of the follicular, unicystic and follicular/desmoplastic variant occurred more in males. The acanthomatous, basal cell, desmoplastic, follicular/acanthomatous and the follicular/acanthomatous/cystic types occurred exclusively in males. Majority of the cases (72, 93%) occurred in the mandible (Fig 1) while the maxilla accounted for only 2 cases (3%) (Table 2). 3 cases (4%) were reported to have occurred in soft tissue with each presenting in the 2^nd^, 3^rd ^and 7^th ^decade of life. The posterior mandible comprising of the body and ramus region were involved in 34 cases whose specific sites were known, whereas the anterior region was involved in only 23 cases (Table 3). However there were considerable overlaps in the sites involved in some cases.

**Table 2:** Distribution of Histological types of Ameloblastoma according location and gender

	**Location**					
**Histological types**	**Mandible**		**Maxilla**		**Soft tissue**	
	**M**	**F**	**M**	**F**	**M**	**F**
Follicular	24	22	1	0	0	3
Plexiform	6	3	1	0	0	0
Acanthomatous	3	0	0	0	0	0
Basal cell	2	0	0	0	0	0
Desmoplastic	4	0	0	0	0	0
Unicystic	0	1	0	0	0	0
Cystic/Follicular	2	1	0	0	0	0
Follicular/Desmoplastic	0	2	0	0	0	0
Follicular/Acanthomatous	1	0	0	0	0	0
Follicular/Acanthomatous/Cystic	1	0	0	0	0	0
**Total**	72(93%)		2(3%)		3(4%)	

**Table 3:** Site distribution of Ameloblastoma of the Mandible

**Site**	**No**	**%**
Anterior Mandible (Incisor-Canine region)	23	40.4
Middle Mandible (Premolar-Molar region)	28	49.1
Posterior Mandible (Ramus region)	6	10.5
		
**Total**	57	100.0

* Only tumours with known specific location on the mandible were considered for analysis

Follicular ameloblastoma, the most prevalent histological type (64.9%) seen show the arrangement of the ameloblastomatous cells in discrete islands, with 46 cases occurring in the mandible and only 1 case in the maxilla. Incidentally all the 3 cases occurring in the soft tissue are follicular in type (Table 2). The mean age of occurrence (± SD) was 28.5 ± 11.2 (range 11–70 years) (Table 4) with the peak incidence in the 3^rd ^decade of life (Table 1). 10 cases (13.0%) demonstrated plexiform arrangement of ameloblastomatous cells with a male to female ratio of 2.3:1 (7 males, 3 females). 9 of the cases occurred in the mandible while the maxilla accounted for only 1 (Table 2). The mean age of occurrence (± SD) was 41.3 ± 20.5 (range 16–70 years) (Table 4) and the peak incidence was in the 3^rd ^decade of life (Table 1). Acanthomatous ameloblastoma showing squamous metaplasia of the cells at the center of the tumour islannds accounted for 3 cases (3.9%) with all occurring exclusively in males and in the mandible (Table 2). The mean age of occurrence (± SD) was 61.3 ± 1.2 (range 60–62 years) (Table 4) with a peak incidence in the 7^th ^decade of life (Table 1). Only 2 cases (2.3%) demonstrated features resembling those of basal cell carcinoma of the skin and were seen exclusively in males and in the mandible and within the 4^th ^decade of life (Tables 1 and 2). Desmoplastic ameloblastoma, accounting for 4 cases (5.2%) occurred only in males and in the mandible (Tables 2). The mean age of occurrence (± SD) was 36.5 ± 4.4 (range 25–39 years) (Table 4) with a peak incidence in the 4^th ^decade of life (Tables 1). A diagnosis of unicystic ameloblastoma was made in 1 case (1.3%) with the ameloblastoma arising from the wall of a unilocular odontogenic cyst. It showed mural proliferation of ameloblastomatous cells and occured in the female and in the mandible and in the 4^th ^decade of life (Tables 1 and 2).

**Table 4:** Analysis of Age of patients (years) according to the Histological type

**Histological type**	**Mean**	**N**	**Std Deviation**	**Minimum**	**Maximum**
Follicular	28.5	50	11.2	11.0	70.0
Plexiform	41.3	10	20.5	16.0	70.0
Acanthomatous	61.3	3	1.2	60.0	62.0
Basal cell	40.0	2	0.0	40.0	40.0
Desmoplastic	36.5	4	4.4	30.0	39.0
Unicystic	40.0	1	0.0	40.0	40.0
Cystic/Follicular	23.7	3	6.5	17.0	30.0
Follicular/Desmoplastic	25.0	2	0.0	25.0	25.0
Follicular/Acanthomatous	45.0	1	0.0	45.0	45.0
Follicular/Acanthomatous/Cystic	21.0	1	0.0	21.0	21.0
**Total**	32.1	77	14.1	11.0	70.0

**Figure 2:**
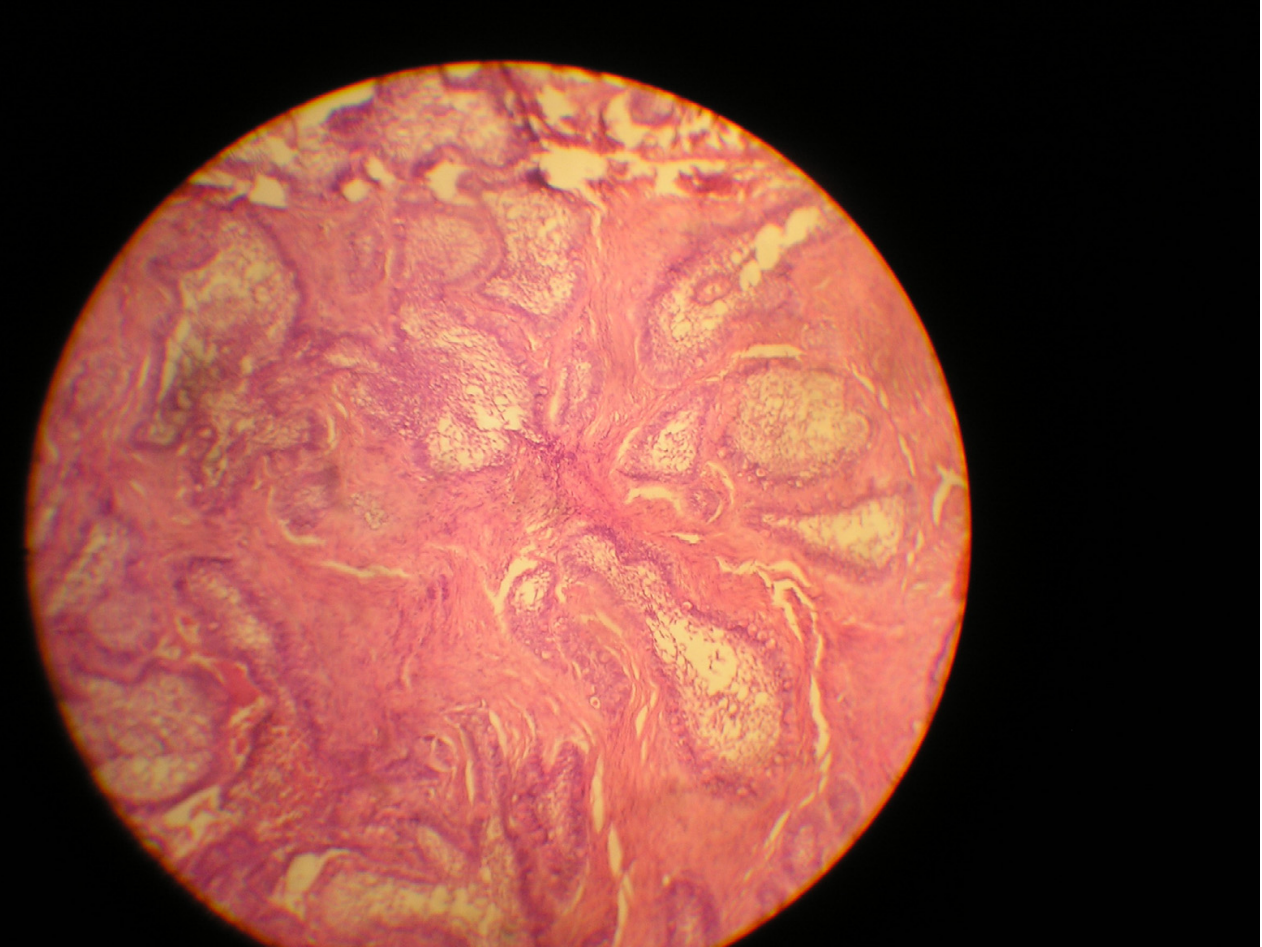
Photomicrograph of follicular ameloblastoma exhibiting desmoplasia of the connective tissue stroma (H&E, 100×).

**Figure 3:**
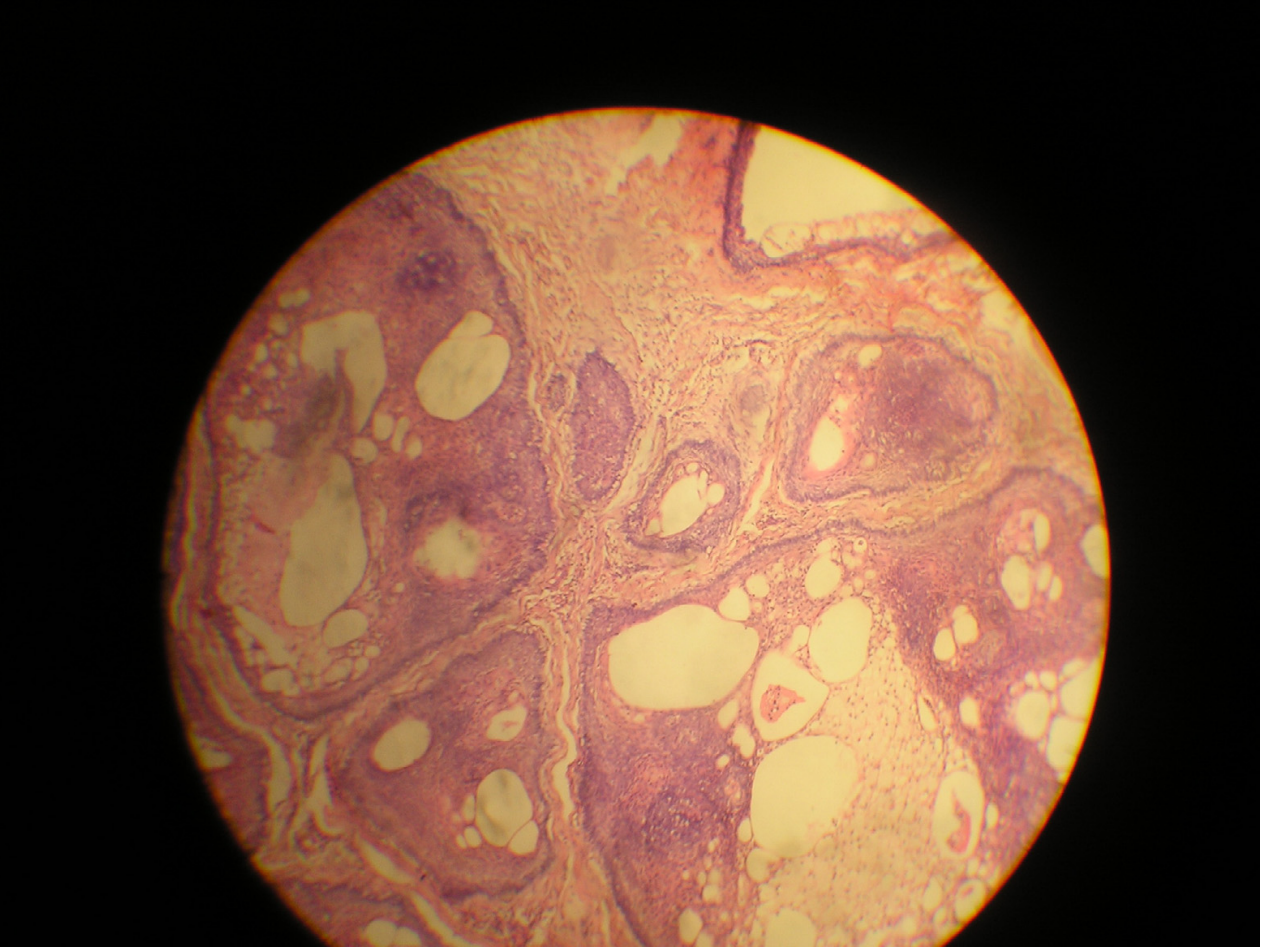
Photomicrograph of follicular ameloblastoma showing cystic degeneration (H&E, 100×).

Combinations of various histological features (Figs 2 and 3) were demonstrated by some of the cases of ameloblastoma reviewed as analysed in Tables 1, 2 and 4.

## Discussion

Generally, odontogenic tumours have been reported to be rare and that it takes considerable time for any center to collect representative cases in sufficient numbers [5]. However, mosadomi [6] reporting that ameloblastoma was the most common jaw tumour in Nigerians claimed that West Africans show a predisposition for ameloblastoma. Though this agrees with other reports from the same region [7,8], it is at variance with findings in Latin America where odontomas were more frequent than ameloblastoma [9,10]. Numerous histological patterns have been described in ameloblastomas. Some may exhibit a single histological subtype; others may display several histological patterns within the same lesion. Common to nearly all subtypes is the polarization of cells around the proliferating nests in a pattern similar to ameloblasts of the enamel organ [11].

Our observation that follicular ameloblastoma is the most prevalent histological variant (64.9%) in the present study agrees with reports in the literature [8,11]. This is followed by the plexiform (13.0%), desmoplastic (5.2%) and acanthomatous (3.9%) varieties (Table 1). It should be noted however that in some cases the assessment of predominant histological pattern is undoubtedly subject to some degree of sampling error since it is well known that large ameloblastomas often show a mixture of several histological patterns. Consequently, an accuracy of assessment with respect to the predominant histological subtype based on small biopsy specimen may be questioned. According to Chapple and Manogue [12], follicular ameloblastoma consists of discrete follicles with a similarity to the stellate reticulum of the enamel organ and with a varying quantity of conjunctive tissue stroma. The covering epithelium is columnar or cuboidal with nuclei positioned opposite the basal membrane. Squamous metaplasia such as that seen in acanthomatous ameloblastoma may be attributed to chronic irritation. Calculus and oral sepsis (which could be a source of chronic irritation) have been suggested to play a role in aetiology of ameloblastoma [13].

In this study the wide age range observed for follicular and plexiform ameloblastomas (11–70 years) compare favourably with the reports in Nigeria [7,8,14] and Korea [15]. The peak age of incidence in the 3^rd ^decade of life is similar to the reports of Ladeinde et al [16] but differs from the peak incidence of 5^th ^decade reported by Waldrom & El-moffy [17]. However our report showed that acanthomatous variant occurred in the 6^th ^and 7^th ^decades. The 4^th ^decade accounted for all the cases of basal cell variant, unicystic variant and three out of the four reported cases of desmoplastic ameloblastoma.

The reported male predilection of ameloblastoma in the literature [14-16] was confirmed by all the histological variants in this series with the exception of the follicular type which showed equal gender distribution and the unicystic and follicular/desmoplastic type which occurred exclusively in females (Table 2). However other reports from Nigeria [6] and elsewhere [2,18] showed equal gender distribution while a female predominance was reported in another series [19].

The mandibular predilection of all the histological variants in this series agrees with reports in the literature [7,16] with only two (3%) (one follicular, one plexiform) out of the 77 cases in our report occurring in the maxilla. The observation that the most common site of occurrence was the middle mandible (premolar-molar region) (Table 3) is consistent with other reports in the literature [8,17,20]. The soft tissue accounted for three (4%) reflecting the relatively low incidence of this extraosseous (peripheral) counterpart of the central ameloblastoma. This low incidence agrees with reports in the literature but its distribution in 2^nd^, 3^rd ^and 7^th ^decades in our series is inconsistent with other reports where the 6^th ^decade was favoured [1,8,21]. However, the low number of reported cases in this series provides little ground for comparison with other studies where over 22 cases were reported [1,21].

There is now more detailed reference to the unicystic variety because it compares favourably with the solid or multicystic counterpart in terms of clinical behaviour and response to treatment [22]. It is a also well known fact that the granular cell variant and ameloblastoma exhibiting clear cell differentiation which were not seen in our series, are more biologically aggressive than other ameloblastomas [23-25], hence the significance of our collected data to the pathologist and clinician

## Conclusion

This study provides a baseline data on variants of ameloblastoma as obtained in a suburban Nigerian population. Since variants of ameloblastoma differ in biologic behaviour, the data collected in this study provides clinicopathologic information which is of significance to the pathologist and clinician

## Competing interests

The author(s) declare that they have no competing interests.

## Authors' contributions

KEA – Has made major contributions to conception and study design. He has been involved in collecting, analysing and interpreting the data.

VIU – Has made substantial contributions to conception and study design and has been involved in revising it critically

GOO – Was involved in collecting the data. She has revised the manuscript critically for important intellectual content.

KCN – Has revised the manuscript critically for important intellectual content.

FOO – Has revised the manuscript critically for important intellectual content.
